# Engineered microbial biosensors based on bacterial two-component systems as synthetic biotechnology platforms in bioremediation and biorefinery

**DOI:** 10.1186/s12934-017-0675-z

**Published:** 2017-04-14

**Authors:** Sambandam Ravikumar, Mary Grace Baylon, Si Jae Park, Jong-il Choi

**Affiliations:** 1 Biomolecules Engineering Lab, Department of Biotechnology and Bioengineering, Chonnam National University, 77 Yongbong-ro, Gwangju, 61186 Republic of Korea; 2 Division of Chemical Engineering and Materials Science, Ewha Womans University, 52 Ewhayeodae-gil, Seodaemun-gu, Seoul, 03760 Republic of Korea

**Keywords:** Two-component regulatory system, Biosensor, Bioremediation, Genetic circuit, Biorefinery

## Abstract

Two-component regulatory systems (TCRSs) mediate cellular response by coupling sensing and regulatory mechanisms. TCRSs are comprised of a histidine kinase (HK), which serves as a sensor, and a response regulator, which regulates expression of the effector gene after being phosphorylated by HK. Using these attributes, bacterial TCRSs can be engineered to design microbial systems for different applications. This review focuses on the current advances in TCRS-based biosensors and on the design of microbial systems for bioremediation and their potential application in biorefinery.

## Background

Toxic chemicals have currently been released into the environment by accidental spills and the improper management of chemical industries. These toxic chemicals include inorganic products such as heavy metals and organic products such as benzene, toluene, ethylbenzene, biphenyl, and styrene, accidental release of which into environment are a significant threat to the environment. Heavy metals and oil products are difficult to remove from the environment and cannot be easily degraded. Thus, they are ultimately indestructible and constitute a global environmental hazard. As a result, soil and groundwater contamination has become a major problem at these polluted sites and requires urgent remediation technology to protect the environment.

Over the past few decades, several technologies based on novel analytical methods have been developed to remove certain metals and organic pollutants from the environment [1]. Unfortunately, many conventional techniques have been found to be ineffective and/or expensive due to low permeability, different subsurface conditions, and contaminant mixtures. Owing to the limitations of traditional methods, researchers have focused on in situ bioremediation, which uses microorganisms to degrade petroleum products or immobilize heavy metal contaminants. Bioremediation strategies have been proposed as potential alternatives for the removal of organic and inorganic pollutants due to their safety, speed, low cost, and high efficiency in removing pollutants from the environment.

**Fig. 1:**
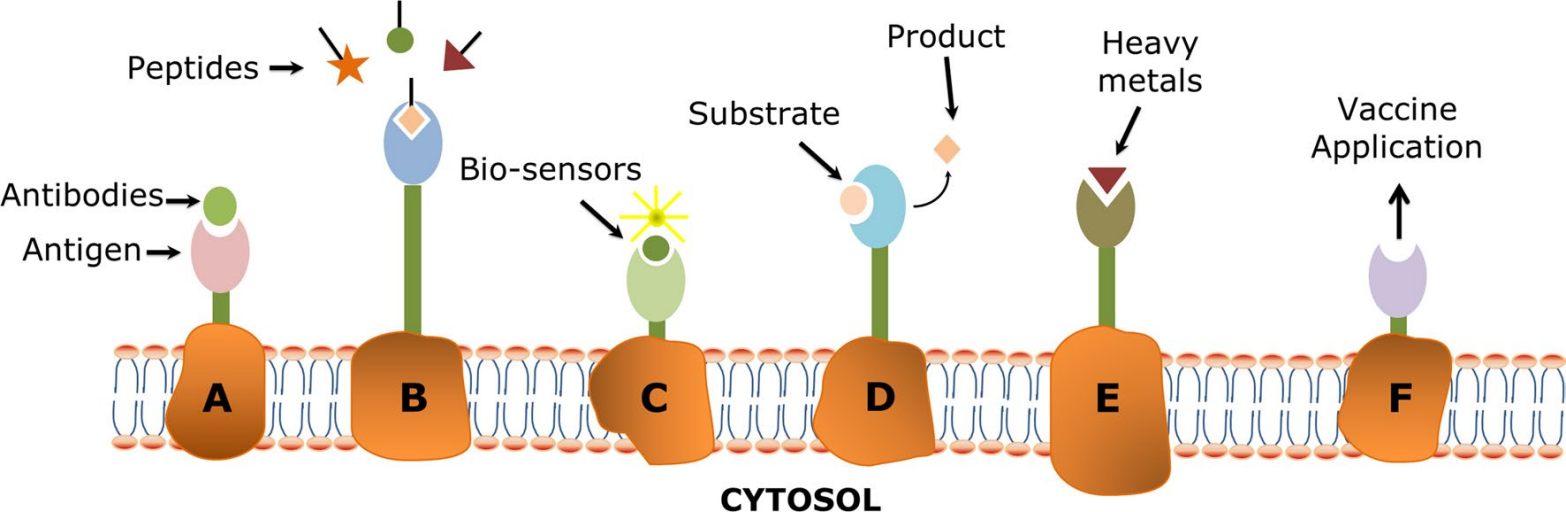
Application of different cell surface display technologies in *A* antibody production, *B* peptide library screening, *C* biosensors, *D* biocatalysts, *E* bio-adsorption, and *F* vaccine development

The central principle of bioremediation is that microorganisms are able to produce energy they need to grow and reproduce by degrading hazardous contaminants. In some cases, bioremediation occurs spontaneously because the essential materials required for bacterial growth are naturally present at the contaminated sites. More often, bioremediation requires an engineered bacterial system to accelerate the tailor-made biodegradation of organic compounds or bio-adsorption of inorganic elements as we desired [2, 3]. It is also needed to further optimize the environmental conditions, in which the microorganisms carry out the detoxification reactions by employing several engineered microorganism systems such as cell surface display- and secretion-based strategies to remediate the contaminated environment. Cell surface display technologies have widely been used in both pharmaceutical and bioremediation applications such as live vaccine development, antibody production, peptide library screening, biosensors, bio-adsorption of organic and inorganic pollutants, and whole-cell biocatalysis (Fig. 1) [4–7].

Heavy metals are common pollutants that are byproducts of various industrial activities. Microorganisms usually mobilize metals from one location and scavenge metals from another. Recently, recombinant bacterial systems displaying chimeric proteins on the cell surface have been developed for use in the bio-adsorption of specific heavy metals. To address organic products, microorganisms have been engineered to produce extracellular enzymes or display enzymes as outer membrane proteins, and they act as a whole-cell catalyst to break down petroleum hydrocarbons and their derivatives. However, all of these constructs require expensive inducers, or the constitutive expression of a membrane protein on the cell surface may affect the growth of the host system. Additionally, none of these engineered bacteria can sense the particular bio-component to be degraded. Therefore, engineered bacteria should be designed to monitor the environmental pollutants, and the design should also include a well-defined removal system. The engineered bacterial system should behave normally until it senses the target in the environment. Once the target is detected, the system should modulate bacterial genes in response. In this way, the genes needed to remove the target are only transcribed and expressed when required. Therefore, it is essential to construct an inexpensive system that can efficiently examine and remove hazardous materials present in the environment.

Nature has provided an excellent solution to this problem. Interestingly, cells have evolved many intricate sensory apparatuses to control cellular growth and behavior. Thus, some cells not only sense light, temperature, oxygen, and pH, but also detect the toxic status of the external environment. An essential requirement for a biosensor or bioremediation process is promoting contact between the contaminants and microbes. As a result of this contact, the microbes adapt their cellular functions in response to the surrounding environmental conditions and then express the relevant genes when needed. If the aim is to monitor and remove an individual toxic compound from the environment, then a synthetic biological strategy will be more feasible because the necessary genetic circuits can be assembled to sense and reduce the level of the exogenous toxin. These synthetic genetic circuits can be assembled using a two-component regulatory system (TCRS) in bacteria [8].

Two-component regulatory systems are widely found in prokaryotes, but only a few have been identified in eukaryotic organisms that can be coupled to environmental stimuli for an appropriate cellular response. This system senses environmental changes and regulates cellular metabolism in response to these changes thereby allowing bacteria to grow, thrive and adapt in different environments. A prototypical TCRS has two components: a histidine kinase (HK) and a response regulator (RR). The HK sensor is a homodimeric integral membrane protein that contains a sensor domain as an extracellular loop located between two membrane-spanning segments (TM1 and TM2) and a transmitter domain located in the last transmembrane segment confined to the cytoplasm. All HK domains contain two highly conserved domains: dimerization and histidine phosphotransfer domain (DHp) and catalytic ATP-binding domain (CA). The periplasmic or extracellular region serves mostly as the signal recognition domain. The DHp and CA domains are responsible for the molecular recognition of the cognate RR as well as the hydrolysis of ATP. The transmitter domain, which serves as a signal transmitter linking the periplasmic and cytoplasmic regions, contains three domains that are named after the proteins where they were first discovered: PAS (Periodic circadian proteins, Aryl hydrocarbon nuclear translocator proteins and Single-minded proteins), HAMP (HKs, Adenylate cyclases, Methyltransferases, and Phosphodiesterases), and GAF (cGMP-specific phosphodiesterases, adenylyl cyclases, and formate hydrogenases). These domains can either transmit signals from the periplasmic region or directly recognize the cytoplasmic signals. Therefore, the HK senses stimuli from the external environment and autophosphorylates conserved histidine residues in the kinase itself. The RR is regulated by the HK, which phosphorylates aspartate residues on the RR. The phosphorylated RR generates output by binding to promoters and thus activates or represses gene expression [8].

Aside from the application of TCRSs in the development of engineered microorganisms for coupled detection and degradation of environmental pollutants, recently, the potential application of TCRSs to metabolically engineered microorganisms has also been extensively examined for different biotechnological purposes. Thus, the recent advances in TCRS-based biosensors designed for cell-mediated bioremediation in response to different environmental pollutants are discussed along with the potential application of TCRSs for the development of engineered host microorganisms in biorefinery process to produce bio-based chemicals.

### TCRS sensing of heavy metals and organic pollutants

**Fig. 2:**
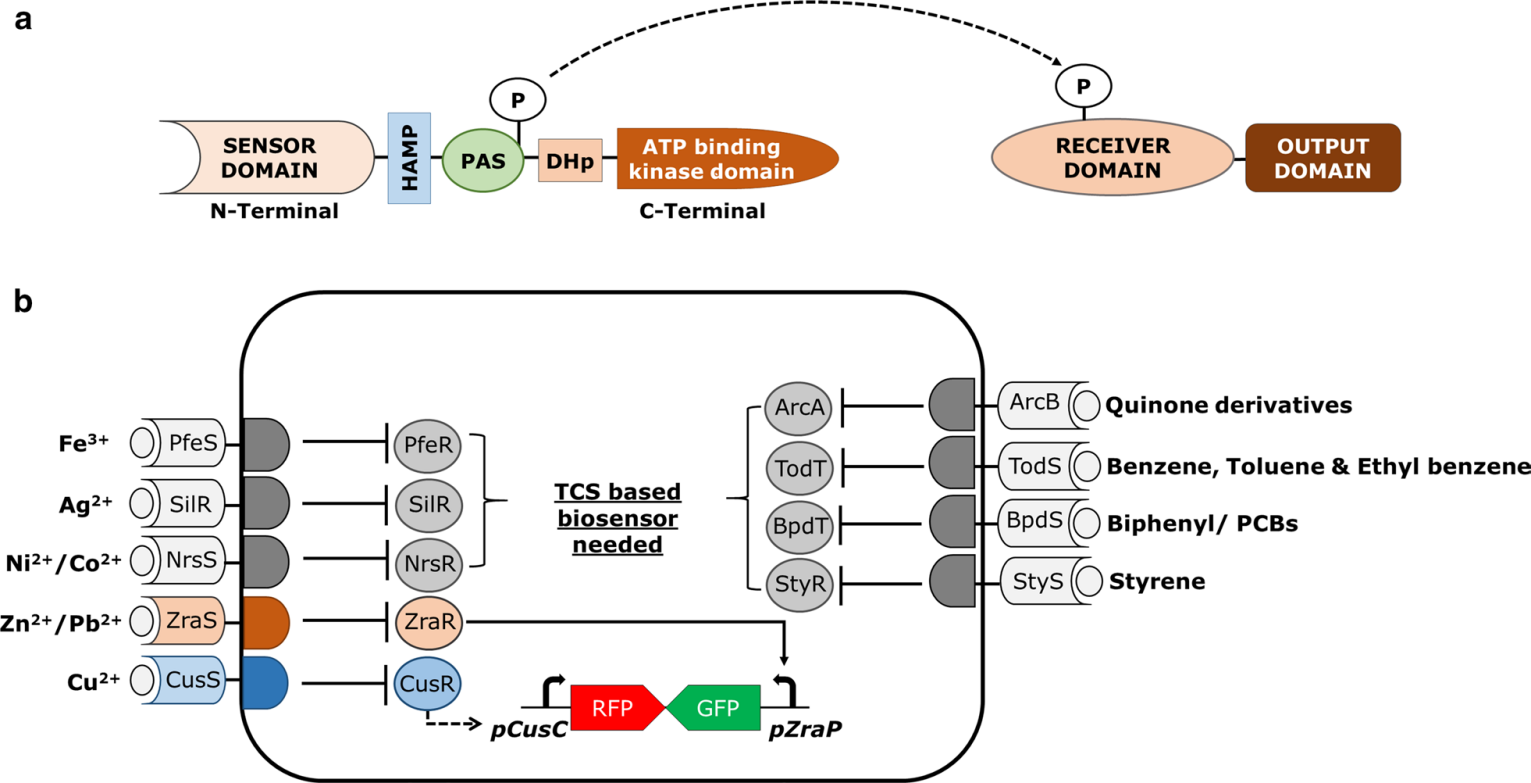
**a** Domain structure of bacterial two-component regulatory systems (TCRS). Typical two-component phosphotransfer systems contain a sensor domain and a cytoplasmic response regulator (RRs). **b** A multi-component phosphorelay system containing the HAMP, PAS, and phosphotransfer domains. The periplasmic metal-sensing receptors sense heavy metals and phosphorylate the HK domain and activate the corresponding RR. The RR activates the synthetic genetic circuit of the TCRS resulting in the expression of the reporter protein. The genetic circuit shown in *gray* can be developed as a biosensor

Two-component regulatory systems can detect a broad range of environmental signals, such us light, oxygen, pH, temperature, and even some heavy metals and organic contaminants [9]. Many types of TCRS-based environmental biosensors have been reported, but only a few heavy metal- and organic pollutant-based sensors have been developed to date (Fig. 2). Bacteria use several TCRSs to sense specific heavy metals. Because heavy metals are cations that are both toxic and essential, bacterial cells use TCRSs to regulate the homeostasis of these metal cations. A HydHG TCRS (also known as ZraSR) was identified in *Escherichia coli* that senses and controls the expression of *zraP* gene encoding zinc efflux protein under high concentrations of Zn^2+^ and Pb^2+^ in aerobic condition [10]. HydH protein is tightly bound to the cell membrane and is assumed to be responsible for sensing high periplasmic Zn^2+^ and Pb^2+^ concentration. Then, in the presence of a phosphoryl donor, HydG binds to the intergenic region within *zraP*-*hydHG* resulting in the upregulated expression of ZraP [10]. Likewise, the CusRS (*ylcA, ybcZ*) TCRS found in *E. coli* K-12 is responsive to Cu^2+^ ions and is required for the inducible expression of *pcoE*, belonging to the plasmid-borne *pco* operon, the induction of the genes in this operon activates the copper efflux system thereby allowing the excess Cu^2+^ to exit the cell [11]. Some TCRS can regulate the expression of several specific genes in an operon or a whole operon. The SilRS TCRS increases the resistance of *Salmonella enterica* to silver cations through the coupled sensing and activation expression of the periplasmic silver-specific binding protein, SilE encoded by *silE* gene and two parallel efflux pumps, SilP and SilCBA [12]. This is also in the case of NrsSR TCRS identified in *Synechocystis* sp. PCC6803. NrsSR senses Ni^2+^ and Co^2+^ ions and regulates the expression of the *nrsBACD* operon that encodes proteins involved in Ni^2+^ resistance [13]. In another study, a PfeS/R TCRS senses ferric enterobactin and induces the production of the enterobactin receptor PfeA in *Pseudomonas aeruginosa* [14].

Aromatic compounds are the most abundant organic contaminants. However, utilizing these compounds is disruptive to most bacteria. Due to the genetic and metabolic flexibility of bacteria, some microorganisms can use organic contaminants as their sole carbon source. Several TCRSs have been identified to be involved in catabolizing aromatic compounds by inducing and activating the aromatic metabolism pathways. The TodST TCRS of *Pseudomonas putida* can be induced by different aromatic substrates such as toluene, xylene, benzene, and ethylbenzene. This TCRS modulates the expression of the *tod* genes, which encode enzymes for the catabolism of these aromatic compounds [15]. The StySR TCRS identified in *Pseudomonas* sp. strain Y2 activates the expression of the *styABCD* genes in response to changes in styrene concentration in the environment [16]. Another TCRS, BpdST, potentially controls biphenyl or polychlorobiphenyl degradation in *Rhodococcus* sp. [15].

### TCRS-based heavy metal bio-adsorption coupled with a biosensor

One of the best approaches to a biosensor-based method is to use a genetically modified microorganism that emits a clear signal when the microbes encounter a target molecule [17, 18]. To date, many metal-specific and a few petroleum product-based bacterial sensors have been developed [19–23]. Based on the nature of the cells used, a variety of TCRS-based environmental contaminant sensors has been constructed by several research groups. However, to remediate environmental pollutants, new synthetic genetic circuits are needed so that the bacterial system can have both sensor and remediation activities. Future research on the application of biosensors in bioremediation should focus on the development of such TCRSs. Some of the TCRS-based heavy metal biosensors for use in bioremediation applications have been developed and are reviewed below.

A zinc adsorption system was developed by using the ZraSR TCRS and chimera Zinc binding OmpC. In normal microbial system, ZraSR detects and induces the membrane protein ZraP, which is responsible for the efflux of Zn^2+^ ions. Engineered zinc adsorption system was based on normal ZraSR TCRS, in which ZraS is used for detecting Zn^2+^ ions, but the ZraR activates the *ompC*-Zinc binding peptide chimeric gene under the ZraP promoter instead of native ZraP. The zinc binding peptides displayed in the cell surface can adsorb exogenous Zinc. This system is sensitive to zinc even at low concentrations (0.001 mM) [24].

In the same manner, simultaneous detection and removal of copper ions in the bacterial surface was achieved through the combined application of CuSR TCRS and cell surface displayed copper binding peptides (CBP) fused to the membrane protein OmpC. In this system, CuSR induces the expression of the chimera OmpC-CBP upon sensing Cu^2+^ ions. Then, the chimera proteins expressed in bacterial cell surface can adsorb the copper ions [25].

An interesting feature of these adsorption systems is that the expression of the chimeric OmpC with the metal binding site is induced by heavy metals (Table 1). Hence, the construction of a heavy metal biosensor in combination with a bio-adsorption system would complement analytical heavy metal detection methodologies and enable the rapid monitoring and removal of toxic levels of bioavailable metal contaminants in industrial settings. The above biosensor combined with bio-adsorption was able to absorb heavy metals efficiently without any induction system. Following this scheme, this synthetic bacterial system is an excellent paradigm for developing multifunctional synthetic systems that can be applied both in the efficient removal and recovery of the target compound.

**Table 1:** Two-component regulatory systems based on microbial biosensors coupled with bio-adsorption

Field of application	TCRS	Function	Host chassis	Promoter-reporter	Chemical target	Detection range (mM)	References
Bioremediation	ZraSR (also known as HydHG)	Biosensor	*E. coli* XL1-blue	*zraP*-*gfp*-HydG	Zinc	0.01–1	[66]
	CuSR	Biosensor	*E. coli* XL1-blue	*cusC*-*gfp*-CusR	Copper	0.004–1	[25]
	ZraSR and CusSR	Biosensor coupled with bio-adsorption	*E. coli* XL1-blue	*zraP*-*gfp, cusC*-*gfp*	Zinc and Copper	0.05–1	[67]
	ZraSR	Biosensor coupled with bio-adsorption	*E. coli* TOP10	*zraP*-*gfp*-*ompC*	Lead	0.3–1	[68]
	ZraSR	Biosensor coupled with bio-adsorption	*E. coli* XL1-blue	*zraP*-*gfp*	Zinc	0.1–1	[24]
Biorefinery	DcuSZ (Chimeric)	Biosensor	*E. coli* BL21 (DE3)	*ompC*-*gfp*	Fumarate	0.1–10	[55]
	MalKZ (Chimeric)	Biosensor	*E. coli* BL21 (DE3)	*ompC*-*gfp*	Malate	0.1–10	[56]
	AauSZ (Chimeric)	Biosensor	*E. coli* BL21 (DE3)	*ompC*-*gfp*	Acidic amino acid	0.05–10	[57]
	Tazl (Chimeric)	Biosensor	*E. coli* RU1012	*ompC*-*lacZ*	Aspartate	0.2–1	[28]

### Engineering chimeric TCRSs for detecting novel compounds

The successful design and construction of TCRS provide a better understanding of the system to obtain a chimeric TCRS customized for achieving a desired input/output. The HK domain, which has a variety of signal recognition capabilities, may be used to couple or shuffle a broad range of input signals to the appropriate output responses through a conserved phosphotransfer process. This shuffling can be achieved by cross-linking the domains of evolutionarily distinct TCRSs, and a chimeric TCRS with the desired sensing ability can be obtained. Most of the domain shuffling required for rational design of chimeric proteins is between HKs and rarely between RRs. At present, several research groups have successfully constructed a chimeric two-component sensor protein by fusing the HK domain to the sensory domain of another kinase or a completely unrelated protein. These studies improve our understanding of the molecular events that occur during signal transduction across membranes in these organisms.

Engineering receptor kinases mainly involve a domain swapping or shuffling strategy in which a receptor protein or another HK contributes their functional module. The domain swapping in HKs implies that these proteins are flexible, allowing the construction of new kinases using a rational design strategy. The domain swapping strategy has been used to produce chimeric TCRSs that include chemotaxis proteins. There are several periplasmic chemotactic receptors, such as Tsr, Tar, Trg, and Aer, that recognize specific chemicals, and they can be coupled with the cytoplasmic domain of EnvZ to allow signal transduction [26]. EnvZ is the most studied HK protein that regulates the phosphorylation state of OmpR in response to osmolarity changes. OmpR is an RR protein responsible for the controlled expression of *ompF* and *ompC* genes encoding for the membrane porin proteins OmpF and OmpC, respectively. Aside from OmpR, EnvZ can also regulate the phosphotransfer of 11 different RRs found in *E. coli* [27]. Because the EnvZ–ompR complex in *E. coli* is a well-studied TCRS that is widely-distributed in bacteria, the DHp and CA domains of EnvZ are commonly used for the domain swapping strategy. A good example of this is the hybridization of Tar, a chemoreceptor transmembrane protein that can detect aspartate and EnvZ. By replacing the cytoplasmic signaling domain of Tar protein with the cytoplasmic kinase/phosphatase domain of EnvZ, the hybridized proteins were able to carry out both the sensing capability of Tar for aspartate and the regulation capability of EnvZ towards OmpR thereby consequently activating *ompC* [28]. This strategy also worked in the hybridization of Trg protein and EnvZ, allowing the recognition of ribose-binding peptides and activation expression of *ompC* [29]. In addition to functioning as chemotactic receptors, HK domains are also involved in light sensing, and kinases that sense C_4_-dicarboxylate, sugar, aspartate, and acidic amino acids have been engineered with the EnvZ cytoplasmic domain to provide a better sensing ability for the desired substance (Table 1). This approach to engineering novel two-component sensor proteins not only acts as a high throughput screening system but also provides knowledge of the newly identified two-component signaling pathways.

### Chimeric TCRS-based screening and regulation of microbial chemical production

In line with the depletion of fossil fuels, renewable biomass is being exploited as a sustainable substitute for petroleum. Among the renewable biomass resources, lignocellulosic biomass is one of the most promising due to its abundance. Lignocellulosic biomass undergoes different pretreatment methods that result in a hydrolysate containing mixed sugars and inhibitors that can be detrimental to the growth of microbial cells during fermentation [30].

**Fig. 3:**
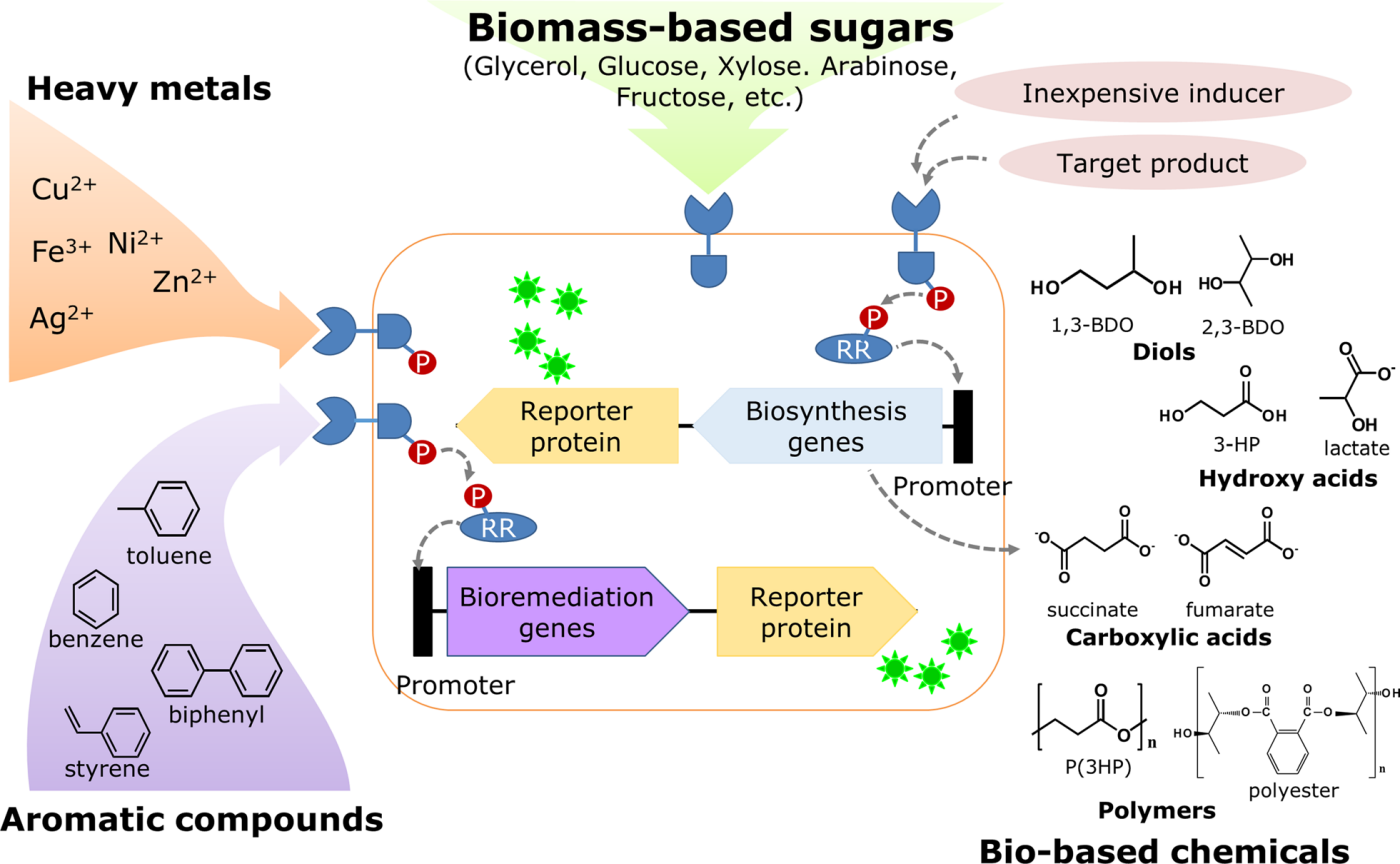
Application of TCRSs in bioremediation and microbial biorefinery. TCRSs serve as a regulatory system for the expression of genes encoding enzymes for the degradation of the detected target pollutant compound or for genes encoding enzymes for the production of the target chemical product

Metabolic engineering strategies have been developed in systems level for the development of metabolically engineered microorganisms as host strains in biorefinery processes to produce bio-based fuels [31–35], chemicals [36–41] and polymers [42–47] from renewable resources. Also, engineered strains that have high levels of growth and tolerance in the presence of high concentrations of sugars and inhibitors are extensively being developed to utilize biomass-derived renewable resources [48–53]. Therefore, it is important to develop a high-throughput screening method to identify the high-producing strains. High-producing strains can be screened using a riboselector, which is composed of a riboswitch that can detect the target compound and a selection module such as *tetA*, which will enable favorable growth of a lysine-accumulating cell in the presence of selection pressure (NiCl_2_) [54]. Likewise, chimeric TCRS can be potentially used in screening for high-producing strains (Fig. 3). DcuSZ is an EnvZ/OmpR-based chimeric TCRS that was constructed by fusing the DcuS HK sensory domain with the cytoplasmic domain of EnvZ. The chimeric DcuSZ is highly specific to fumarate in such a way that the expression of the *gfp* gene under the control of the *ompR*-regulated *ompC* promoter is proportional to different fumarate concentrations in the medium [55]. Other chimeric TCRSs based on EnvZ/OmpR were constructed by fusing the HK sensory domain of MalK and AauS to the EnvZ catalytic domain to detect high malate- and aspartate-producing strains, respectively [56, 57].

Two-component regulatory systems may also be used to develop tightly regulated gene expression systems. Tightly regulated gene expression is important in engineering metabolic pathways to avoid leaky expression that may cause a metabolic burden to the microbial cell. Typical induction strategies include the use of isopropyl-β-d-thiogalactopyranoside (IPTG). However, IPTG is expensive and can be toxic to cells at high concentrations. An example of tightly regulated gene expression induced by an inexpensive substrate is the invertible promoter system. In this system, the promoter is active or ‘ON’ when the target substrate that serves as an inducer is present and ‘OFF’ (inverted orientation) when absent [58]. Based on this invertible promoter system’s mechanism, the coupled sensing and regulating activities of TCRSs can be modified to achieve tightly regulated gene expression.

### Summary and perspectives

To date, some TCRSs have been identified that sense organic compounds (benzene, toluene, ethylbenzene, biphenyl, styrene, fumarate, and malate) and regulate the gene expression of proteins involved in catabolic pathways. These compounds can be metabolized and used as a carbon source for most groups of microorganisms [9]. In TCRSs, the signal recognized by the sensor kinase domain catalyzes the ATP-dependent phosphorylation of a conserved histidine residue in the protein. The phosphoryl group is then transferred from the histidine to an aspartate residue located in the RR. The phosphorylated RR binds to specific promoter sequences to either activate or repress transcription. At present, a wide range of synthetic genetic circuits has been developed that can couple a sensor output to a desired biological activity [59]. In addition, numerous genetic switches are also available to turn on gene expression once a target molecule has reached its activation threshold. A switch can be assembled using transcriptional repressors or activators, which allows the connection between the sensor output and regulation of the biological response [58]. Several switch types have been developed to control the cellular response: inverter switches that produce a reciprocal response [60]; biphasic switches that use both negative and positive regulation and respond to small amounts of input [61]; toggle switches that use two repressors that cross-regulate each other’s promoters [62]; and riboswitches that regulate gene expression by inhibiting protein synthesis [63]. Likewise, many logic gate types have been developed for biological circuits, including ‘NAND’, ‘NOT IF’ and ‘NOR’ [64].

**Fig. 4:**
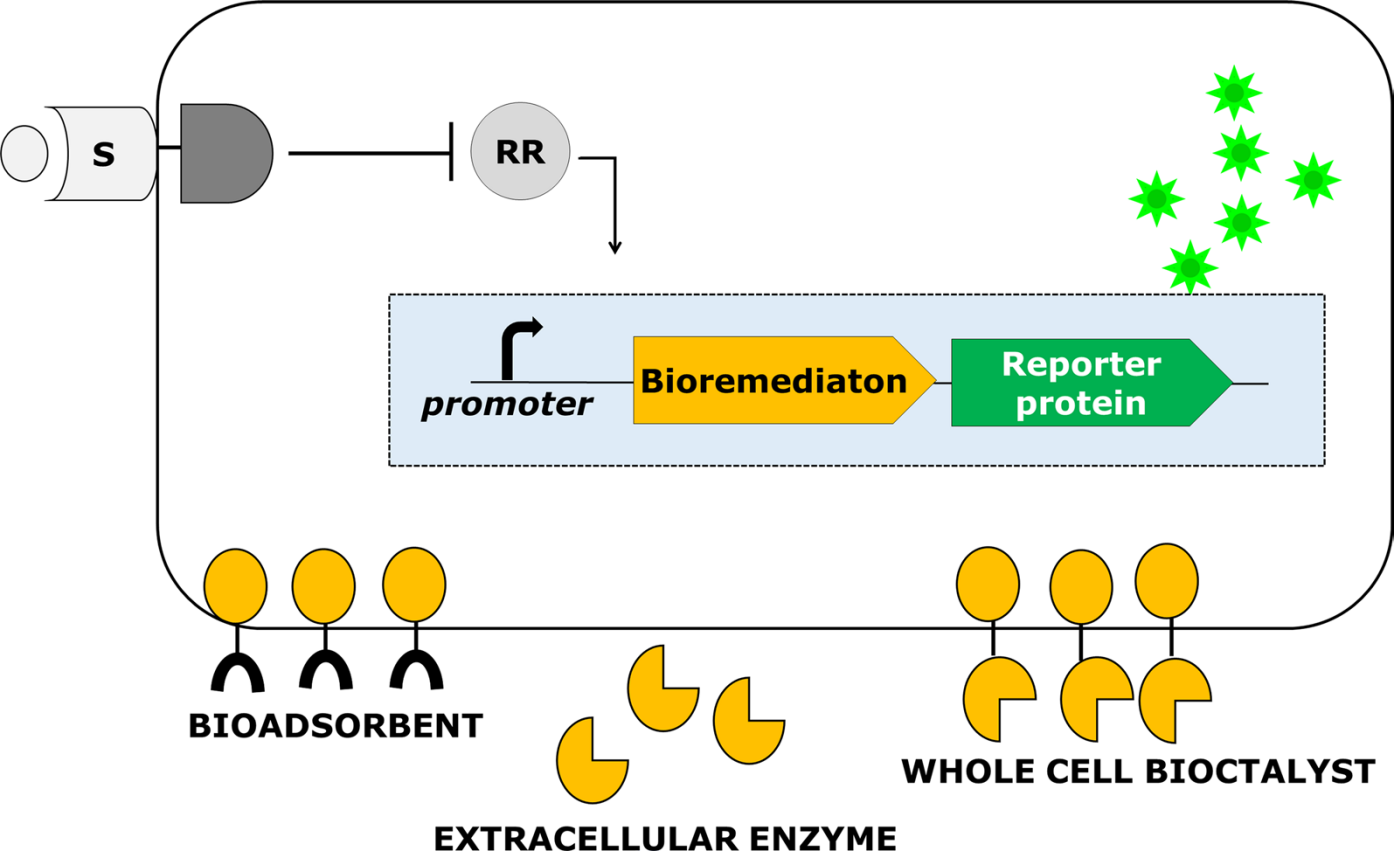
Synthetic TCRS with integrated biosensing and bioremediating functions for the detection of the target compound and upregulation of the effector protein that allow real-time detection of controlled gene expression

Integrated approaches provide a better perspective for developing a specific biosensor designed to catalyze the production and/or degradation of the desired compound. To achieve this, it is necessary to rewire the genetic circuits of bacteria using the above synthetic devices. Design of the engineered system should be based on strategies for building sensory regulation components that incorporate a target substrate-responsive TCRS in any desired host (Fig. 4). Introducing a sensory regulation device in a host cell enables it to sense the target compound and trigger the genetic circuit, achieving real-time monitoring of the compound present and upregulation of the effector protein’s gene expression. Use of engineered TCRSs in bacteria would prevent the production of redundant proteins at the initial growth phase and avoid the use of toxic and costly chemical inducers.

Although a large number of accessible sensor parts are available for TCRSs, employing these sensors in a domain shuffling strategy can be challenging. To attach the sensor domain to the HK domain of the protein, structural and functional information on both proteins is needed [65]. When designing chimeric TCRS-based biosensors, great care is required in domain swapping to maximize the kinase activity of the chimeric protein. In the majority of the chimeric TCRS-based biosensors, monitoring of the extracellular targets and the response to these targets is achieved by producing a reporter protein [55–57]. Moreover, biosensors have also been modified with other synthetic biology tools such as the bio-absorption of heavy metals with a cell surface display system and expression of an extracellular enzyme to degrade aromatic compounds. Therefore, such a synthetic genetic circuit can be switched on when a signal is detected to remove certain pollutants, and after the input signal disappears, the microbes behave like normal bacteria.

## Conclusions

In this review, we have discussed numerous TCRSs engineered in different prokaryotic species that can sense inorganic and organic pollutants, and examined the recent developments in cellular biosensors coupled with bioremediation. The TCRS-based biosensor coupled with bioremediation approach has the potential to advance even further using the recent developments in bioengineering in strain development. However, only a few studies on TCRS-based biosensors have been reported, and much effort is needed to obtain a complete picture of the TCRS-based control of downstream catabolic pathways. To achieve these goals, a thorough understanding of TCRS mechanisms is essential to engineer strains for use in efficient biosensor systems coupled with bio-degradation or bio-adsorption functionality. Moreover, more studies are required to extend its use in food, pharmaceutical and industrial biotechnology applications.

## Abbreviations

TCRS: two-component regulatory system
HK: histidine kinase
RR: response regulator
DHp: histidine phosphotransfer domain
CA: catalytic ATP-binding domain
ATP: adenosine triphosphate
